# Association Between Maternal Dietary Isoflavone Intake During Pregnancy and Childhood Allergic Rhinoconjunctivitis: The Japan Environment and Children’s Study

**DOI:** 10.3390/nu17050769

**Published:** 2025-02-21

**Authors:** Gui Yang, Aya Hisada, Midori Yamamoto, Rieko Takatani, Yuki Konno, Chisato Mori, Kenichi Sakurai, The Japan Environment and Children’s Study (JECS) Group

**Affiliations:** 1 Department of Nutrition and Metabolic Medicine, Center for Preventive Medical Sciences, Chiba University, 1-33 Yayoicho, Inageku, Chiba 263-8522, Japan; ki-you@chiba-u.jp (G.Y.);; 2 Department of Sustainable Health Science, Center for Preventive Medical Sciences, Chiba University, 1-33 Yayoicho, Inageku, Chiba 263-8522, Japan; midoriy@faculty.chiba-u.jp (M.Y.);; 3 Department of Environmental Preventive Medicine (Yamada Bee Company, Inc.), Center for Preventive Medical Sciences, Chiba University, 1-33 Yayoicho, Inageku, Chiba 263-8522, Japan; 4 Department of Bioenvironmental Medicine, Graduate School of Medicine, Chiba University, 1-8-1 Inohana, Chuo-ku, Chiba 260-8670, Japan

**Keywords:** maternal nutrition, isoflavone intake, allergic rhinoconjunctivitis, birth cohort study

## Abstract

**Background/Objectives:** Isoflavone (ISO) may have immunosuppressive and promoting effects. In this study, we aimed to examine the association between maternal dietary ISO intake during pregnancy and childhood allergic rhinoconjunctivitis at the age of 3 years using the Japanese Birth Cohort data. **Methods**: Overall, 78,549 mother–child pairs were studied. Maternal dietary ISO intake (the sum of genistein and daidzein) during pregnancy was evaluated using a food frequency questionnaire. Information on physician-diagnosed allergic rhinoconjunctivitis was collected from the caregiver-reported questionnaire. After classifying ISO intake into quartiles (Q1: reference), multivariable logistic regression analysis was employed to explore the association with allergic rhinoconjunctivitis. **Results**: No association was observed between maternal ISO intake and allergic rhinoconjunctivitis in any child. However, in the sex-stratified analysis, maternal ISO intake linked to allergic rhinoconjunctivitis in female children positively (Q2, adjusted odds ratio [aOR]: 1.22, 95% confidence interval [CI]: 1.06–1.40; Q3, aOR: 1.17, 95% CI: 1.01–1.35; Q4, aOR: 1.24, 95% CI: 1.07–1.44). **Conclusions**: Maternal dietary ISO intake during pregnancy was sex-specifically linked to childhood allergic rhinoconjunctivitis in children. These findings provide insights into the need for estimating the optimal ISO consumption during pregnancy for allergy avoidance in children.

## 1. Introduction

Over the last two decades, the widespread prevalence of childhood allergies, particularly allergic rhinitis and conjunctivitis, has dramatically increased [1,2]. Allergic rhinitis and conjunctivitis are prevalent chronic inflammatory diseases with similar pathophysiological processes, characterized by nasal congestion, rhinorrhea, sneezing, and itchy, watery eyes. Accordingly, they are collectively referred to as allergic rhinoconjunctivitis [3], which typically co-occurs with other conditions that can lower the quality of life, such as sleep disturbances and mental health illness [4,5]. Immune system abnormalities cause allergic rhinoconjunctivitis, and its onset is linked to a complex combination of genetic and environmental factors [4,6]. Immune cells, such as T lymphocytes, are detected in the thymus between 9 and 14 weeks of pregnancy [7,8], whereas the precursors of B cells are found as early as 7 weeks in the fetal liver [9]. However, the immune system continues to develop throughout the fetal period [10].

Nutrition throughout pregnancy and early childhood markedly impacts fetal health status, influencing epigenetic programming and modulating immune cells and cytokines, which can have some impacts on the development of allergic diseases at later stages of life [11,12,13,14,15]. For instance, a maternal diet containing eggs and raw vegetables during pregnancy may decrease the risk of having childhood allergic rhinitis, whereas meat consumption may increase this risk [16]. Moreover, prenatal maternal vitamin D consumption may reduce the possibility of having childhood allergic rhinitis [17], while a higher prenatal maternal dietary content of *n*-6:*n*-3 fatty acids can increase the likelihood of this [18].

Isoflavone (ISO) is a kind of phytoestrogen having an estrogen-related chemical structure. Depending on the levels of endogenous estrogen, ISO can perform as an estrogen agonist or antagonist, as well as exert anti-inflammatory, antioxidant, and tyrosine kinase inhibitory properties [19,20,21]. ISO is abundant in soybean and soy products, predominantly in ISO glycosides. These ISO glycosides are converted into aglycones of genistein (GEN), daidzein (DAI), and glycitein by salivary and small intestine mucosal enzymes, or β-glucosidase produced by intestinal bacteria and subsequently absorbed through the digestive tract [22,23]. Additionally, GEN and DAI can be carried to the fetus via the placenta, with fetal blood concentrations being higher than maternal concentrations in humans [24]. Thus, an essential research focus includes exploring the association between maternal ISO intake during pregnancy and the health outcomes of children.

Some in vivo studies focusing on the long-lasting consequences of ISO exposure have revealed that prenatal maternal exposure to ISO may have impacts on the immune system of their offspring [25,26,27,28,29,30]. Moreover, maternal ISO intake during pregnancy may have immunosuppressive effects on offspring [27,28]. Additionally, administering GEN to pregnant mice may have immune-activating effects only in female offspring, not male offspring [29,30]. In summary, maternal ISO intake during pregnancy may either promote or inhibit the development of childhood allergic diseases. Therefore, the subject of interest is whether maternal ISO intake during pregnancy is associated with the development of childhood allergic diseases positively or negatively and whether this effect differs depending on the sex of the child.

To our knowledge, the association between maternal dietary ISO intake during pregnancy and childhood allergic diseases in humans has not yet been well established. Moreover, the incidence of childhood allergic rhinitis keeps rising and represents one of the possible risk factors for asthma [5]. In the current study, we aimed to evaluate a large birth cohort to assess the association between maternal dietary ISO intake during pregnancy and childhood allergic rhinoconjunctivitis, along with assessing any potential sex-specific effects of this association.

## 2. Materials and Methods

### 2.1. Study Design

We analyzed a dataset acquired from the Japan Environment and Children’s Study (JECS), which is a nationwide government-funded birth cohort study, and recruited expectant mothers from 15 Regional Centers between January 2011 and March 2014 [31,32]. The JECS is aimed to assess the effects of environmental exposure during pregnancy and early childhood stages on the health and development of children. The JECS protocol was reviewed and approved by the Ministry of the Environment’s Institutional Review Board on Epidemiological Studies (Approval Number: No. 100910001), as well as the ethics committees of all the participating institutions. The JECS was carried out in compliance with the Declaration of Helsinki, and all participants gave written informed consent.

### 2.2. Study Population

**Figure 1:**
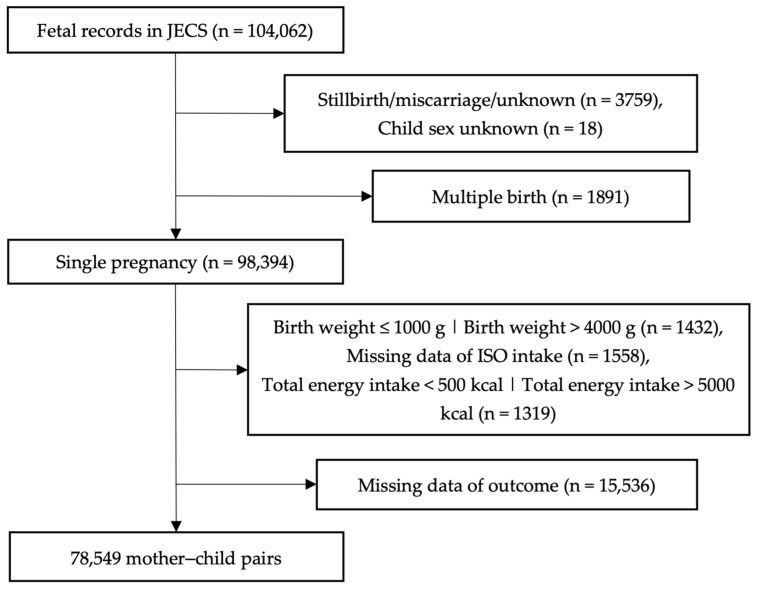
Participant flow diagram.

The present research relied on the “jecs-ta-20190930” dataset, which included 104,062 fetal records in October 2019. We excluded cases of miscarriage, stillbirth, missing birth information (*n* = 3759), unknown child sex (*n* = 18), multiple pregnancies (*n* = 1891), extremely high (>4000 g) or low (≤1000 g) birth weight (*n* = 1432), missing ISO intake data (*n* = 1558), and extremely high (>5000 kcal) or low (<500 kcal) total energy intake (*n* = 1319). Finally, we analyzed 78,549 mother–child pairs after excluding missing data of outcome (*n* = 15,536) in the study (Figure 1).

### 2.3. Exposure

Maternal ISO intake was used as an exposure variable. Data from the self-administered food frequency questionnaire (FFQ) were used to estimate the maternal ISO intake during pregnancy. The FFQ was answered during mid/late pregnancy to evaluate the maternal nutritional intake in the past month. The FFQ assesses the frequency of food intake and serving size of different food items [33], and it appears that the following 10 food items are linked with ISO intake: natto (fermented soybeans), tofu for miso soup, tofu for other dishes, yushidofu (re-drained tofu), koyadofu (freeze-dried tofu), atsuage (thick, deep-fried tofu), aburaage (deep-fried tofu), miso soup, soy milk, and kinako (roasted soy flour). Tofu, natto, and miso accounted for >80% of ISO intake. The daily intake of GEN and DAI was calculated with a specifically developed food composition table for the ISO in Japanese food [34]. The GEN and DAI estimated from the FFQ were confirmed to be correlated to those estimated from the 12-day weighted food record (*r* = 0.53; *r* = 0.55) [33]. Additionally, given that GEN and DAI account for most ISO intake [35,36], the total GEN and DAI intakes were applied to represent ISO intake.

### 2.4. Outcome

The outcome variable was allergic rhinoconjunctivitis, as diagnosed by physicians at the age of 3 years. Information regarding diagnosis was collected from the caregiver-reported questionnaires, which asked: “Has your child ever had an immune system disorder diagnosed after the age of 2 years?: allergic rhinitis (including pollinosis-induced rhinitis), 0 = no, 1 = yes, allergic conjunctivitis (including pollinosis-induced conjunctivitis), 0 = no, 1 = yes”. We offered a definition of allergic rhinoconjunctivitis as a positive response to either of these two questions.

### 2.5. Covariates

According to the body of knowledge relating to the risk factors for allergy diseases in children [5,6,37], we created directed acyclic graphs to determine the associated covariates using the online software DAGitty version 3.1, developed and maintained by Johannes Textor (Institute for Computing and Information Sciences, Radboud University and Medical BioSciences Department, Radboud UMC, Nijmegen, The Netherlands) [38]. We then used the following factors to modify the multivariable analysis: maternal age at delivery (continuous variable), maternal educational level (≤12 years, >12 years), maternal smoking status (never smoker, ex-smoker who quit before pregnancy, ex-smoker who quit during pregnancy, current smoker), pre-pregnancy body mass index (<18.5 kg/m^2^, 18.5–25 kg/m^2^, ≤25 kg/m^2^), maternal history of allergies (no, yes), maternal blood folic acid concentration during mid/late pregnancy (continuous variable), total energy intake (continuous variable), and nutritional condition of children up to 4 months of age (breastfeeding, formula milk, mixed). Further, in order to account for the influence of fruits and vegetables on this association [39], we adjusted the effects of fruit intake and vegetable intake as covariates.

### 2.6. Statistical Analyses

The social and demographic characteristics of the individuals were displayed using descriptive statistics. The median and interquartile ranges were given for all of the continuous variables that were not normally distributed, including ISO intake, maternal blood folic acid concentration, and total energy intake. Means and standard deviations were presented for maternal age, whereas amounts, as well as percentages, were applied for the remaining variables that were categorical. Multivariable logistic regression analysis was utilized to figure out the odds ratios and 95% confidence intervals (CIs) for the association between maternal ISO intake during pregnancy and childhood allergic rhinoconjunctivitis at the age of 3 years. The Food Safety Commission of Japan recommends a safe upper limit for ISO intake of 70–75 mg/day; however, pregnant women, children, and fetuses have not been included in the discussion of this value [40]. Therefore, given the non-normal distribution and lack of cutoff values for recommended ISO intake in pregnant women, we classified ISO intake into categorical variables by quartiles (Q1: reference, Q2, Q3, Q4). For each covariate, the missing values were less than 1%; thus, the complete data were analyzed. In the stratified analysis, the sex of the child was used as a stratification factor.

In addition, sensitivity analyses were performed to verify the potency of the association between maternal ISO intake during pregnancy and childhood allergic rhinoconjunctivitis. The first sensitivity analysis was performed using a model with fruit intake and vegetable intake added as adjusted variables to account for the influence of fruits and vegetables. Next, we used multiple imputations using the chained equations algorithm in sensitivity analyses to complement the missing covariate values. Ten complete datasets containing exposure, outcomes, and covariates were created, and a sequence of appropriate replacements was applied to all of the missing covariate values. We then performed multivariable logistic regression and stratified analyses according to child sex using the multiple imputation data set. Lastly, considering that childhood allergic diseases are strongly influenced by maternal allergy history, we further ran a subgroup analysis to control the effects of maternal allergy history.

We performed all statistical analyses using R version 4.2.0 (Institute for Statistics and Mathematics, Vienna, Austria; www.r-project.org, accessed on 1 September 2022).

## 3. Results

### 3.1. Participant Characteristics

Table 1 shows the characteristics of the involved mothers and their children according to the ISO intake quartile. Of the 78,549 children, 51.1% were male and 48.9% were female. The prevalence of childhood allergic rhinoconjunctivitis was 5.5%, while 58.3% of the mothers had a history of allergies. The mean maternal ISO intake was 30.39 mg/day.

### 3.2. Association Between Maternal ISO Intake and Childhood Allergic Rhinoconjunctivitis

Univariate and multivariate analyses of the association between maternal ISO intake during pregnancy and childhood allergic rhinoconjunctivitis at the age of 3 years and stratified analysis by child sex are presented in Table 2. Maternal ISO intake during pregnancy was associated with childhood allergic rhinoconjunctivitis (crude odds ratio [cOR]: 1.11, 95% CI: 1.02–1.21) in the crude model; however, this association disappeared after adjusting the effects of confounding factors (aOR: 1.09, 95% CI: 0.99–1.20). The sex-stratified analysis revealed that maternal ISO intake associated positively with allergic rhinoconjunctivitis in female children (Q2, aOR: 1.22, 95% CI: 1.06–1.40; Q3, aOR: 1.17, 95% CI: 1.01–1.35; and Q4, aOR: 1.24, 95% CI: 1.07–1.44, respectively) but not in male children.

### 3.3. Sensitivity Analysis

In the first sensitivity analysis, we observed an increased risk in female children after adjusting for the additional fruit intake and vegetable intake (Table S1). In addition, the multiple imputation data set analysis results were comparable to those of the complete data set (Table S2). According to the findings of the subgroup analysis of mothers with and without a history of allergies (Tables S3 and S4), maternal ISO intake was associated positively with allergic rhinoconjunctivitis only in female children, regardless of maternal allergy history.

## 4. Discussion

The association between maternal ISO intake during pregnancy and allergic rhinoconjunctivitis in offspring was evaluated in this study. Maternal ISO intake during pregnancy was positively connected with childhood allergic rhinoconjunctivitis only in female children. Our findings are suggestive of the potential involvement of maternal ISO exposure in the development of allergic rhinoconjunctivitis after birth in female offspring.

ISO has an estrogen-like structure and a binding affinity for estrogen receptors [41]. Estrogen receptors are broadly located in immune cells [42], and estrogen promotes the T_H_2 immunological response [43], which also links to the balance of T_H_1/T_H_2. Additionally, skewed T_H_2 immunological response is associated with a higher risk of allergic diseases. In other words, ISO may increase the risk of allergic diseases via the Th2 immune response. In partial support of this hypothesis, our data suggest that maternal ISO intake during pregnancy may associate with an increased risk of allergic rhinoconjunctivitis in the offspring. In particular, pregnant women have been found to exhibit a T_H_2-biased immunological response throughout pregnancy, and newborns exhibit skewed T_H_2 immunological responses [44,45]. After birth, a gradual switch to the T_H_1 immunological response occurs as “immune-maturing” while maintaining a balanced T_H_2 immunological response [44]. However, infants who cannot maintain a healthy balance retain T_H_2 immunological predominance and develop allergies [45,46]. Moreover, maternal ISO exposure may increase maternal and fetal serum estrogen levels [47]. In mice, ISO exposure in utero interacting with estrogen receptors may increase serum total IgE in both female and male adult offspring [48]. Therefore, maternal ISO intake may strengthen the T_H_2 immunological response of the offspring. Thus, we considered that the increased estrogen levels caused by fetal ISO exposure, together with the estrogenic effect of ISO crossed via the placenta, may promote or maintain T_H_2 immunity, increasing the risk of allergic rhinoconjunctivitis in children.

Together with the T_H_1/T_H_2 balance, eosinophils also play a role in the pathophysiology of allergic rhinitis. Estrogen also affects eosinophil function and contributes to the onset of allergic rhinitis. Individuals with allergic rhinitis have eosinophils in their nasal mucosa, and eosinophils degranulate in their nasal secretions [49,50]. Estrogen increases eosinophil adhesion to the mucosa and mucosal vessels, as well as eosinophil degranulation [51]. Thus, increased estrogen levels associated with maternal ISO intake may also promote eosinophil adherence and degranulation in the nasal mucosal vessels. This possibility may partially explain the increased risk of allergic rhinoconjunctivitis development in the offspring of mothers with high ISO intake.

Additionally, previous studies have reported that gut microbiota is related to allergic disease [52], while maternal gut microbiota during pregnancy is also associated with the development of childhood allergic disease in their offspring [53,54]. Furthermore, ISO may affect intestinal microbiota [55,56]. Therefore, it is possible that maternal ISO exposure may have also affected the development of allergic disease in the child by altering the maternal intestinal microbiota during pregnancy.

Epidemiologically, male children have more serum-specific immunoglobulins E (IgE) than female children [57], as well as a higher prevalence of allergic rhinitis [58]. Consistent with a previous report, our study identified a higher prevalence of allergic rhinoconjunctivitis in male children than in female children (6.1% vs. 4.7%). However, in our sex-stratified analysis, the association between maternal ISO intake during pregnancy and allergic rhinoconjunctivitis was observed only in female children, not male children. It has further been reported that fetal exposure to nutrients and environmental chemicals has different effects on male and female offspring owing to sexual dimorphism [59,60]. Epidemiological data have indicated that exposure to bisphenol A, which has a chemical structure similar to the estrogen-like ISO, during pregnancy may increase the risk of childhood allergic diseases only in female children [61]. This sex specificity may be explained by the differences in the amount of estrogen receptors by sex [62]. In fact, animal studies have reported that female mice have higher levels of estrogen in their amniotic fluid than males [63], and female human fetuses have higher amniotic fluid GEN and DAI levels than male fetuses [64]. Furthermore, as previously documented, ISO metabolism shows sex differences [65,66,67], while GEN and DAI have longer pharmacokinetics half-lives in females than in males [68]. Thus, the differences in the amount of estrogen receptors in immune cells and the levels of estrogen and ISO may partially explain the sex specificity. In addition, maternal ISO exposure during pregnancy reduces IFN-γ, which impacts IL-12 production, and is associated with T_H_2 immune-promoting consequences in rat female offspring but not in rat male offspring [28]. Oral administration of 20 mg/kg GEN to pregnant mice reportedly promotes IL-4 synthesis only in female offspring [29]. Specifically, continuous GEN exposure from gestational day 14 to postnatal day 84 increases blood IgE levels in female offspring [30]. These animal studies further support the sex-specific findings of our study. Although the precise mechanisms remain unknown, existing research suggests that chemicals with estrogenic activity may have sex-specific effects on allergy development in children. Our findings contribute to this body of evidence, highlighting potential differences in susceptibility based on sex.

There are two strengths in this study. This is, to the best of our knowledge, the first study to demonstrate an association between maternal ISO intake during pregnancy and childhood allergic rhinoconjunctivitis in humans. Second, we analyzed data from a nationwide Japanese birth cohort, making the findings highly representative of the Japanese population [32]. In addition to these two strengths, our study had certain limitations. First, the findings of this study cannot be utilized to determine optimal ISO consumption for allergy prevention because we could not assess another ISO, glycitein, although it represents only a small proportion of ISO intake. Furthermore, this may have also stemmed from the fact that we used the FFQ to assess ISO intake, which has the risk of underreporting ISO intake [69]. Second, the conclusions drawn from this study are limited to populations with a high ISO intake because the intake levels (mean ± standard deviation) shown in this study (1.54–102.76 mg/day) are higher than data from other countries (0.11–4.24 mg/day) [70]. Hence, caution should be exercised when generalizing the present results. However, our data may be particularly relevant to Asian populations that consume soybeans and soy-based foods, as well as health-conscious individuals and vegetarians who rely on protein sources, such as tofu and soy meat, and have similar levels of ISO intake as the Japanese population [71,72]. Therefore, this information may be beneficial to these individuals as well. Third, the outcome was defined as allergic rhinoconjunctivitis diagnosed by physicians at the age of 3 years; however, information regarding the diagnosis was collected from the reports of caregivers via a questionnaire. It is, therefore, possible that the caregiver-reported diagnosis may have misinterpreted the diagnosis or misremembered information from previous diagnoses. Thus, it is, therefore, possible that recall bias might have affected the accuracy of the results. Fourth, unadjusted confounding variables may have affected our results and data interpretation. Fifth, although children’s diet may be influenced by the maternal diet [73], the children’s consumption of ISO after birth may be associated with allergic rhinoconjunctivitis; however, information on the diets of the children was not collected in the JECS. As a result, we were unable to analyze the children’s diet. Therefore, additional research is necessary to assess the effects of the diets of the children. Sixth, we only investigated the association with allergic rhinoconjunctivitis; additional research on food allergies, atopic dermatitis, and other childhood allergic diseases is required. Seventh, we used the total GEN and DAI intake as the ISO intake index; however, we were unable to assess equol intake using the FFQ. Equol is a DAI metabolite with more significant estrogenic, antioxidant, and anti-inflammatory properties than GEN and DAI [74,75]. Consequently, the metabolic capacity of equol should be considered in future studies using biological samples. Additionally, we were unable to evaluate intermediate factors (e.g., changes in gut microbiota [53,54] or estrogen levels [76]) that could provide a mechanistic explanation for the allergic effects of ISO exposure. Therefore, further research is needed to determine the effect of factors mediated by nutrients consumed during pregnancy. Finally, given the aforementioned limitations, the current findings were unable to provide sufficient evidence for the appropriate consumption of ISO-containing foods during pregnancy.

## 5. Conclusions

Our findings indicated that maternal dietary ISO intake during pregnancy associates positively with childhood allergic rhinoconjunctivitis at the age of 3 years, as well as in a sex-specific manner. However, our findings provide insights into the need for estimating the optimal ISO consumption during pregnancy for allergy avoidance in children. Further studies on ISO and its metabolite equol using biological samples are required.

## Figures and Tables

**Table 1:** Characteristics of the study participants according to ISO intake.

	Quartile for ISO Intake				
	Total	Q1	Q2	Q3	Q4
	*n =* 78,549	*n* = 19,638	*n* = 19,647	*n* = 19,630	*n* = 19,634
ISO (median [IQR]), mg/day	23.36 [13.35–39.65]	7.95 [5.0–10.7]	17.91 [15.6–20.5]	30.02 [26.5–34.5]	55.93 [46.6–70.7]
Maternal age, mean (SD), years	31.0 (4.9)	30.6 (5.1)	31.3 (4.9)	31.7 (4.8)	32.1 (4.7)
Child sex, female (%)	38,400 (48.9)	9579 (48.8)	9634 (49.0)	9538 (48.6)	9649 (49.1)
Maternal educational level, >12 years, *n* (%)	51,843 (66.2)	11,989 (61.3)	12,891 (65.8)	13,301 (68.0)	13,662 (69.8)
Maternal smoking status, *n* (%)					
Never smoker	46,925 (60.1)	11,314 (58.0)	11,897 (60.9)	11,807 (60.5)	11,907 (61.1)
Ex-smoker who quit before pregnancy	18,691 (24.0)	4352 (22.3)	4543 (23.3)	4827 (24.8)	4969 (25.5)
Ex-smoker who quit during pregnancy	9581 (12.3)	2854 (14.6)	2364 (12.1)	2268 (11.6)	2095 (10.8)
Current smoker	2824 (3.6)	980 (5.0)	726 (3.7)	601 (3.1)	517 (2.7)
Pre-pregnancy BMI (kg/m^2^), *n* (%)					
<18.5 kg/m^2^	12,788 (16.3)	3180 (16.2)	3301 (16.8)	3176 (16.2)	3131 (16.0)
≥18.5 and <25 kg/m^2^	58,075 (74.0)	14,288 (72.8)	14,413 (73.4)	14,657 (74.7)	14,717 (75.0)
≥25 kg/m^2^	7646 (9.7)	2155 (11.0)	1923 (9.8)	1791 (9.1)	1777 (9.1)
Maternal history of allergies, yes, *n* (%)	45,639 (58.3)	11,132 (56.9)	11,416 (58.3)	11,588 (59.2)	11,503 (58.8)
Blood folic acid concentration (median [IQR])	6.0 [4.2–10.0]	5.3 [3.7–8.6]	5.8 [4.1–9.5]	6.2 [4.3–10.0]	6.9 [4.7–11.5]
Total energy intake (median [IQR])	1618 [1315–2005]	1375 [1110–1692]	1540 [1285–1859]	1685 [1408–2028]	1909 [1568–2350]
Fruit intake, g/day (median [IQR])	148.7 [95.5–223.4]	102.5 [62.5–156.8]	134.0 [90.6–193.2]	163.3 [113.3–231.9]	208.1 [142.3–303.7]
Vegetable intake, g/day (median [IQR])	108.5 [48.3–192.0]	76.8 [27.2–147.8]	100.5 [46.5–174.2]	118.5 [56.8–198.1]	145.7 [71.9–247.8]
Nutritional condition up to 4 months of age, *n* (%)					
Breastfeeding	33,596 (43.7)	7992 (41.7)	8377 (43.5)	8472 (44.1)	8755 (45.5)
Formula milk	1696 (2.2)	543 (2.8)	397 (2.1)	375 (2.0)	381 (2.0)
Mixed	41,588 (54.1)	10,618 (55.4)	10,493 (54.5)	10,376 (54.0)	10,101 (52.5)
Allergic rhinoconjunctivitis at the age of 3 years, *n* (%)	4298 (5.5)	1029 (5.2)	1063 (5.4)	1068 (5.4)	1138 (5.8)

ISO, isoflavone; quartiles for ISO intake (Q1, Q2, Q3, Q4); IQR, interquartile range; SD, standard deviation; BMI, body mass index.

**Table 2:** Association between maternal ISO intake and childhood allergic rhinoconjunctivitis.

ISO Intake	Total		Male Children		Female Children	
	cOR (95% CI)	aOR (95% CI)	cOR (95% CI)	aOR (95% CI)	cOR (95% CI)	aOR (95% CI)
Q1	1 (reference)	1 (reference)	1 (reference)	1 (reference)	1 (reference)	1 (reference)
Q2	1.03 (0.95–1.13)	1.03 (0.94–1.13)	0.94 (0.84–1.06)	0.92 (0.81–1.03)	1.17 (1.03–1.34)	1.22 (1.06–1.40)
Q3	1.04 (0.95–1.14)	1.04 (0.94–1.14)	0.99 (0.88–1.11)	0.95 (0.84–1.07)	1.11 (0.97–1.28)	1.17 (1.01–1.35)
Q4	1.11 (1.02–1.21)	1.09 (0.99–1.20)	1.06 (0.95–1.19)	1.00 (0.88–1.13)	1.19 (1.04–1.36)	1.24 (1.07–1.44)

ISO, isoflavone; cOR, Crude odds ratio; aOR, Adjusted odds ratio; CI, confidence interval. Adjusted for maternal age at delivery, maternal educational level, maternal smoking status, pre-pregnancy BMI, maternal history of allergies, maternal blood folic acid concentration, total energy intake, and nutritional condition up to 4 months of age.

## Data Availability

The data are not appropriate for public deposition due to ethical limitations and the Japanese legal framework. The public deposition of personal information data is prohibited by the Act on the Protection of Personal Information (Act No. 57 of 30 May 2003, revised 9 September 2015). The open sharing of epidemiological data is similarly restricted by Ethical Guidelines for Medical and Health Research Involving Human Subjects, enforced by the Japan Ministry of Education, Culture, Sports, Science and Technology and the Ministry of Health, Labour and Welfare. For any inquiries about data access, please contact Shoji F. Nakayama: jecs-en@nies.go.jp, the National Institute for Environmental Studies’ JECS Program Office.

## Supplementary Materials

The following supporting information can be downloaded at: https://www.mdpi.com/article/10.3390/nu17050769/s1, Table S1: Association between maternal ISO intake and childhood allergic rhinoconjunctivitis. Table S2: Association between maternal ISO intake and childhood allergic rhinoconjunctivitis using the multiple imputation data set; Table S3: Association between maternal ISO intake and childhood allergic rhinoconjunctivitis in mothers with allergy history; Table S4: Association between maternal ISO intake and childhood allergic rhinoconjunctivitis in mothers without allergy history.

## Abbreviations

aOR	Adjusted odds ratio
BMI	Body mass index
CI	Confidence interval
cOR	Crude odds ratio
DAI	Daidzein
GEN	Genistein
FFQ	Food frequency questionnaire
JECS	Japan Environment and Children’s Study
ISO	Isoflavone
