# Supplementary material for: Association Between Maternal Dietary Isoflavone Intake During Pregnancy and Childhood Allergic Rhinoconjunctivitis: The Japan Environment and Children’s Study
Source: Nutrients. 2025 Feb 21;17(5):769. doi: 10.3390/nu17050769 (PMC11901469; doi:10.3390/nu17050769)
Supplement: Supplementary file 1 [file nutrients-17-00769-s001.zip › nutrients-3451432-supplementary.pdf]

Supplementary Materials

Association between Maternal Dietary Isoflavone Intake During Pregnancy and Childhood Allergic Rhinoconjunctivitis: The Japan Environment and Children's Study

Table S1. Association between maternal ISO intake and childhood allergic rhinoconjunctivitis

ISO	Total		Male children		Female children	
intake	cOR (95% CI)	aOR (95% CI)	cOR (95% CI)	aOR (95% CI)	cOR (95% CI)	aOR (95% CI)
Q1	1 (reference)	1 (reference)	1 (reference)	1 (reference)	1 (reference)	1 (reference)
Q2	1.03 (0.95–1.13)	1.03(0.94-1.13)	0.94 (0.84–1.06)	0.91(0.81-1.03)	1.17 (1.03–1.34)	1.21(1.05-1.40)
Q3	1.04 (0.95–1.14)	1.03(0.94-1.13)	0.99 (0.88–1.11)	0.94(0.83-1.06)	1.11 (0.97–1.28)	1.16(1.01-1.35)
Q4	1.11 (1.02–1.21)	1.08(0.98-1.19)	1.06 (0.95–1.19)	0.98(0.86-1.11)	1.19 (1.04–1.36)	1.23(1.06-1.43)

ISO, isoflavone; cOR, Crude odds ratio; aOR, Adjusted odds ratio; CI, confidence interval,
Adjusted for maternal age at delivery, maternal educational level, maternal smoking status, pre-pregnancy BMI, maternal blood folic acid concentration, total energy intake, fruit intake, vegetable intake, and nutritional condition up to 4 months of age.

Table S2. Association between maternal ISO intake and childhood allergic rhinoconjunctivitis using the multiple imputation data set

ISO intake	Total		Male children		Female children	
	**cOR (95% CI)**	**aOR (95% CI)**	**cOR (95% CI)**	**aOR (95% CI)**	**cOR (95% CI)**	**aOR (95% CI)**
Q1	1 (reference)	1 (reference)	1 (reference)	1 (reference)	1 (reference)	1 (reference)
Q2	1.03 (0.95–1.13)	1.03 (0.94–1.13)	0.94 (0.84–1.06)	0.93 (0.83–1.05)	1.17 (1.03–1.34)	1.18 (1.03–1.35)
Q3	1.04 (0.95–1.14)	1.02 (0.93–1.12)	0.99 (0.88–1.11)	0.95 (0.84–1.07)	1.11 (0.97–1.28)	1.13 (0.98–1.29)
Q4	1.11 (1.02–1.21)	1.08 (0.99–1.19)	1.06 (0.95–1.19)	1.00 (0.88–1.13)	1.19 (1.04–1.36)	1.21 (1.05–1.39)

ISO, isoflavone; cOR, Crude odds ratio; aOR, Adjusted odds ratio; CI, confidence interval
Adjusted for maternal age at delivery, maternal educational level, maternal smoking status, pre-pregnancy BMI, maternal history of allergies, maternal blood folic acid concentration, total energy intake, and nutritional condition up to 4 months of age.

Table S3. Association between maternal ISO intake and childhood allergic rhinoconjunctivitis in mothers with allergy history

ISO intake	Total		Male children		Female children	
	cOR (95% CI)	aOR (95% CI)	cOR (95% CI)	aOR (95% CI)	cOR (95% CI)	aOR (95% CI)
Q1	1 (reference)	1 (reference)	1 (reference)	1 (reference)	1 (reference)	1 (reference)
Q2	1.03 (0.92–1.15)	1.04 (0.93–1.17)	0.92 (0.79–1.07)	0.92 (0.79–1.07)	1.18 (1.00–1.39)	1.23 (1.03–1.45)
Q3	1.05 (0.94–1.17)	1.05 (0.93–1.17)	1.01 (0.87–1.16)	0.99 (0.86–1.16)	1.09 (0.92–1.29)	1.12 (0.94–1.33)
Q4	1.10 (0.99–1.23)	1.08 (0.96–1.22)	1.05 (0.91–1.21)	1.00 (0.85–1.17)	1.18 (1.00–1.40)	1.20 (1.00–1.44)

ISO, isoflavone; cOR, Crude odds ratio; aOR, Adjusted odds ratio; CI, confidence interval
Adjusted for maternal age at delivery, maternal educational level, maternal smoking status, pre-pregnancy BMI, maternal blood folic acid concentration, total energy intake, and nutritional condition up to 4 months of age.

Table S4. Association between maternal ISO intake and childhood allergic rhinoconjunctivitis in mothers without allergy history

ISO intake	Total		Male children		Female children	
	**cOR (95% CI)**	**aOR (95% CI)**	**cOR (95% CI)**	**aOR (95% CI)**	**cOR (95% CI)**	**aOR (95% CI)**
Q1	1 (reference)	1 (reference)	1 (reference)	1 (reference)	1 (reference)	1 (reference)
Q2	0.95 (0.79–1.14)	0.97 (0.80–1.18)	0.86 (0.68–1.09)	0.84 (0.66–1.07)	1.11 (0.83–1.5)	1.23 (0.90–1.67)
Q3	0.98 (0.82–1.18)	0.99 (0.82–1.21)	0.85 (0.67–1.09)	0.80 (0.62–1.03)	1.22 (0.91–1.62)	1.37 (1.01–1.87)
Q4	1.11 (0.92–1.32)	1.13 (0.93–1.38)	0.99 (0.79–1.25)	0.98 (0.76–1.26)	1.32 (0.99–1.75)	1.45 (1.05–1.99)

ISO, isoflavone; cOR, Crude odds ratio; aOR, Adjusted odds ratio; CI, confidence interval
Adjusted for maternal age at delivery, maternal educational level, maternal smoking status, pre-pregnancy BMI, maternal blood folic acid concentration, total energy intake, and nutritional condition up to 4 months of age.
